# Young glaucoma specialist practice patterns: Why do you do what you do?

**DOI:** 10.1016/j.aopr.2025.07.001

**Published:** 2025-07-04

**Authors:** Nicole Miranda, Jason Y. Zhang, Mary Qiu

**Affiliations:** a Pritzker School of Medicine, University of Chicago, Chicago, IL, USA; b Department of Ophthalmology and Visual Science, University of Chicago, Chicago, IL, USA; c Cole Eye Institute, Cleveland Clinic, OH, USA

**Keywords:** Glaucoma, Trabeculectomy, Training experience, Current practice patterns, Survey study

## Abstract

**Background:**

Despite trabeculectomy having long been considered the gold standard surgery for treating severe or recalcitrant glaucoma, the popularity of this operation among glaucoma specialists has gradually declined in recent decades with a concurrent rise in alternative intraocular pressure (IOP)-lowering procedures being performed.

**Purpose:**

This pilot study investigates how experiences of recently graduated glaucoma specialists during training might have influenced their choice to either perform trabeculectomy or favor alternative procedures in their current practice.

**Methods:**

An anonymous questionnaire was distributed to American Glaucoma Society members who completed fellowship training between 2018 and 2022. Questionnaire items assessed respondents' experiences in residency and fellowship training as well as their current practice as attendings regarding four procedures: trabeculectomy, tube-shunt, XEN Gel Stent, and ab-interno angle procedures.

**Results:**

Of 66 total respondents, 64 (97.0%) reported feeling somewhat or entirely comfortable performing trabeculectomy following training. However, 42 (63.6%) said they do not often perform trabeculectomy, whereas 13 (19.7%) reported that they do. Those who reported performing trabeculectomy often (N ​= ​13) were influenced by the surgery's high success rates (92.3%), preoperative (76.9%) and intraoperative (84.6%) processes, and low postoperative complications (61.5%). Those who reported not performing trabeculectomy often (N ​= ​42) were most discouraged by the postoperative process of trabeculectomy, as well as socioeconomic (66.7%) and sociocultural (52.4%) characteristics of their patients. Factors such as volume performed during training (47.6%), cost-effectiveness (19.0%), and reimbursement rates (19.0%) were not considerable contributors.

**Conclusions:**

While most young glaucoma specialists in our study report being comfortable performing trabeculectomy, the majority stated that they do not often perform the procedure in practice. Low trabeculectomy surgical volume during training was not cited as a notable barrier, whereas patient factors and preference for alternative procedures, depending on the clinical scenario, appeared to drive this trend.

## 1 Introduction

Trabeculectomy has long been considered the gold standard intraocular pressure (IOP)-lowering surgery for patients with severe or recalcitrant glaucoma.^1^^,^^2^ Despite this perception, the popularity of trabeculectomies among glaucoma specialists has gradually declined in recent decades. Since the mid-1990s, alternative procedures have emerged with more favorable safety profiles, including certain varieties of tube shunt drainage devices, the XEN Gel Stent (Allergan Inc., CA, USA), and angle-based minimally invasive glaucoma surgeries (MIGS).^3^^,^^4^

The rise in popularity of tube shunts was initially seen following the Tubes Versus Trabeculectomy Study in the late 2000s.^5^ A series of surveys (1996, 2002, 2008, 2016) of American Glaucoma Society (AGS) members, an examination of 1995–2004 Medicare claims data, and an analysis of surgical case logs from 2010 to 2014 ophthalmology residents and 2009–2013 glaucoma fellows all reveal that the use of tube shunts has increased and that of trabeculectomy has declined during this time frame.^3^^,^^6^, ^7^, ^8^, ^9^, ^10^ Meanwhile, since the mid-2010s, angle-based MIGS have also gained popularity due to their effectiveness in managing IOP in appropriate patients with relatively lower risk compared to traditional glaucoma procedures.^11^^,^^12^ Nonetheless, trabeculectomies continue to be considered the gold standard IOP-lowering surgery for patients with severe or recalcitrant glaucoma.^13^

There is growing discussion among more established glaucoma specialists that the shift in practice from trabeculectomies toward alternative procedures may result in junior glaucoma specialists lacking the training to adequately perform trabeculectomies.^14^ In an editorial titled "The Trabeculectomy Must Survive!", Singh et al. discuss that "a decline in trabeculectomy surgery … has the potential to create a downward spiral where less training results in poor results, followed by further reduction in the number of procedures performed, leading to further decrease in training."^11^

Our recent 2023 survey of a sample of 132 AGS members revealed that the proportion of respondents who reported that trabeculectomy was their most preferred traditional glaucoma procedure was 62% among those who completed fellowship more than 15 years ago and 26% among those who completed fellowship within the past 5 years.^15^ Accordingly, the proportion of respondents who reported performing non-valved tubes more frequently than trabeculectomy was 23% among those who completed fellowship more than 15 years ago and 61% among those who completed fellowship within the past 5 years.

Although there is ample evidence that the popularity of trabeculectomy is declining and that of tube shunts and MIGS is rising, there is a paucity of literature regarding how one's experience during residency and fellowship training or any number of other clinical and non-clinical factors might influence a young glaucoma specialist's practice pattern as an attending physician. The purpose of this pilot survey study is to examine how complex factors influence practice patterns among young glaucoma specialists who completed glaucoma fellowship in the United States within the past 5 years.

## 2 Methods

### 2.1 Participants

In this cross-sectional survey study, we developed an anonymous online questionnaire (Supplemental Digital Content 1) that assesses both the training history and current practice patterns of glaucoma specialists who had recently completed their fellowship training in the United States. Eligible participants included glaucoma specialists who graduated from glaucoma fellowship between 2018 and 2022, with 302 individuals qualifying for graduation during this time frame.^16^ This study was deemed exempt from further review by the Institutional Review Board of the University of Chicago (IRB 22–1716). The study adhered to the Tenets of the Declaration of Helsinki.

### 2.2 Questionnaire distribution and design

The questionnaire was developed on the encrypted online platform, Research Electronic Data Capture (REDCap, University of Chicago Secure Server), and distributed to glaucoma specialists by email through the AGS email listserv. Glaucoma fellowship program directors were also contacted via a separate AGS email listserv and asked to share the questionnaire with their recent program graduates. The questionnaire was available to eligible participants from January 2, 2023 to January 7, 2023.

Demographic information was collected from each respondent, including year of graduation, practice setting, and geographic region. The questionnaire then asked a series of questions regarding four different surgical procedures used to manage primary open-angle glaucoma (POAG): trabeculectomy, tube-shunt procedure, XEN Gel Stent procedure (ab-interno or externo), and ab-interno angle procedures. For each procedure, respondents were asked to quantify the number that they had performed as primary surgeon during each phase of their training (residency, fellowship) and the estimated frequency they perform on a monthly and yearly basis as an attending physician. Respondents were then asked to rate their self-perceived level of comfort in performing each surgical technique (yes comfortable, not comfortable, or somewhat comfortable). The respondents were also asked to describe their self-perceived case volume for each procedure as either "high", "low", or "neutral" according to how many they feel that they perform currently as attending physicians. This categorization was not strictly defined to better capture respondents' subjective perceptions. This was elucidated by phrasing the question in our instrument to ask for the respondent's perceived caseload in relation to alternative treatment options. Those who responded with "low" or "neutral" for a given procedure were asked to select which of the other three procedures they would most likely choose instead given a particular clinical scenario.

Next, respondents were presented with a series of statements that reflected possible factors that may have influenced their reported comfort level, with respondents indicating their level of agreement with each statement on a five-point Likert scale. Respondents were considered to be influenced by a factor if they selected "somewhat agree" or "strongly agree" for the provided statement. An optional text box was also provided for further elaboration.

Finally, participants were asked about their opinions on the training requirements for trabeculectomy for ophthalmology residents and glaucoma fellows by selecting the degree to which they agree with the statement "I believe that graduating ophthalmology residents should be required to perform a minimum number of trabeculectomies as primary surgeon." Similarly, participants were asked whether they agree that only glaucoma fellowship-trained surgeons should be expected to feel comfortable performing trabeculectomies (as opposed to comprehensive ophthalmologists without additional fellowship training).

### 2.3 Statistical analysis

Descriptive statistics including mean, interquartile ranges, and frequency were derived using Microsoft Excel 2019 (Microsoft Corp., Redmond, WA). These were used to evaluate survey responses for relationships between respondents' training experiences and variance in their current surgical practices. Associations between specific practice tendencies and demographics were also determined, with statistical significance being assessed using chi-squared testing.

## 3 Results

We received 66 completed questionnaires (response rate 22%) from individuals who completed glaucoma fellowship in the United States between 2018 and 2022 after a five-month period of data collection. The majority of respondents work in either or both academic (43.9%) and/or private practice (59.1%) settings and are practicing in urban (63.6%) and/or suburban (48.5%) settings (Table 1). Notably, there was a statistically significant (*P* ​= ​0.015) higher average number of tube shunts performed each year by providers at academic centers (63.2) compared to private practices (35.6). No correlations were noted with other procedures. No correlations were noted in any procedure when stratified by region (urban vs. suburban).

**Table 1:** Characteristics of the 66 young glaucoma specialists who participated in the survey.

Survey Responses	Number of Responses (%)
Year Graduated	
2018	6 (9.1)
2019	15 (22.7)
2020	15 (22.7)
2021	10 (15.2)
2022	20 (30.3)
Practice Setting	
Academic	29 (43.9)
Private Practice	39 (59.1)
Veteran Affairs	4 (6.1)
County	1 (1.5)
Other	2 (3.0)
Practice Region	
Urban	42 (63.6)
Suburban	32 (48.5)
Rural	6 (9.1)
Other	1 (1.5)

Table 2 shows the median and range of the number of trabeculectomies, tube shunt procedures, XEN Gel Stent procedures, and ab-interno angle procedures performed by the young glaucoma specialists who completed the questionnaire during their residency and fellowship training. The median number of procedures performed in total during residency and fellowship training among our respondents for each surgery type were 29 trabeculectomies (±20.62), 61 tube shunt procedures (±43.20), 5.5 XEN Gel Stent procedures (±17.83), and 69 ​ab-interno angle procedures (±59.04).

**Table 2:** Number of each glaucoma procedure performed during residency and fellowship training (N ​= ​66).^a^

		Percentile				
		0	25	50 (Median)	75	100
**Residency**	**Trabeculectomy**	0	0	2	5	22
	**Tube Shunt**	0	5	8	15	42
	**XEN**	0	0	0	0	20
	**Ab-Interno Angle Procedure**	0	5	10	15	100

**Fellowship**	**Trabeculectomy**	0	13	27	39	80
	**Tube Shunt**	2	35	50	65	200
	**XEN**	0	0	5	15	60
	**Ab-Interno Angle Procedure**	0	40	50	100	200

**Residency and Fellowship**	**Trabeculectomy**	0	15	29	42	102
	**Tube Shunt**	13	41	61	80	220
	**XEN**	0	0	6	19	60
	**Ab-Interno Angle Procedure**	0	43	67	114	250

a Values were rounded to the nearest whole number.

**Fig. 1:**
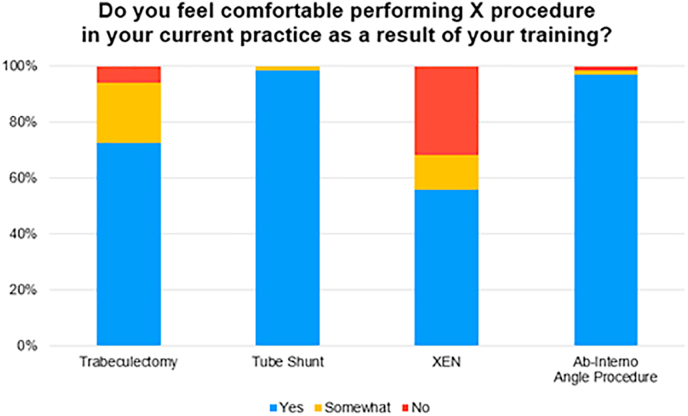
**Self-reported comfort level with each type of glaucoma procedure.** The percentage of respondents who self-reported feeling comfortable performing trabeculectomies, tube shunts, XEN Gel Stents, and ab-interno angle procedures in their attending practice is 73%, 99%, 56%, and 97%, respectively.

Fig. 1 depicts the self-perceived comfort level of respondents in performing each of the four procedures as attending physicians given their experiences during training. While a large majority of respondents reported feeling comfortable or somewhat comfortable performing trabeculectomies (97.0%), tube shunt procedures (100%), and ab-interno procedures (98.5%), only 68.2% of respondents reported a similar level of comfort with XEN Gel Stent procedures (Fig. 1).

**Fig. 2:**
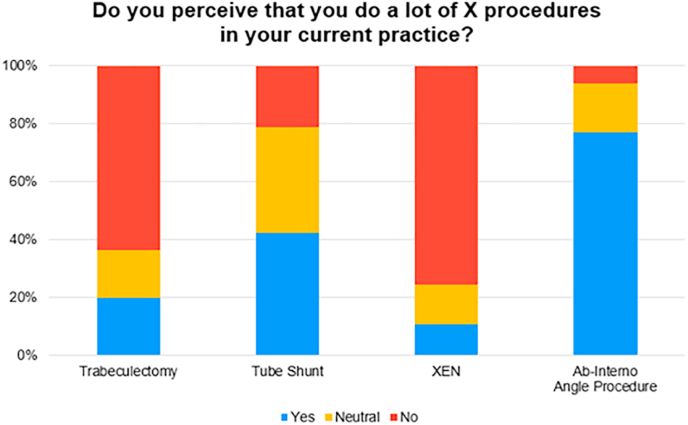
**Self-reported perceived frequency of each type of glaucoma procedure in attending practice**. The percentage of respondents who self-reported that they perceive that they do perform trabeculectomies, tube shunts, XEN Gel Stents, and ab-interno angle procedures often in their attending practice is 20%, 42%, 11%, and 77%, respectively.

Fig. 2 details respondents' perceived surgical volume for each procedure as they perform them in their current practice. Despite most respondents feeling comfortable or somewhat comfortable performing trabeculectomies, as noted in Fig. 1, only 19.7% perceive that they perform a lot of these procedures in their current attending practice, with another 16.7% feeling neutral about the amount of trabeculectomies they perform in their current attending practice. Comparatively, among respondents who feel comfortable performing ab-interno procedures, 75.6% answered *yes* and another 16.7% answered *neutral* to the question of whether they perceive that they perform a lot of ab-interno procedures in their current practice.

**Fig. 3:**
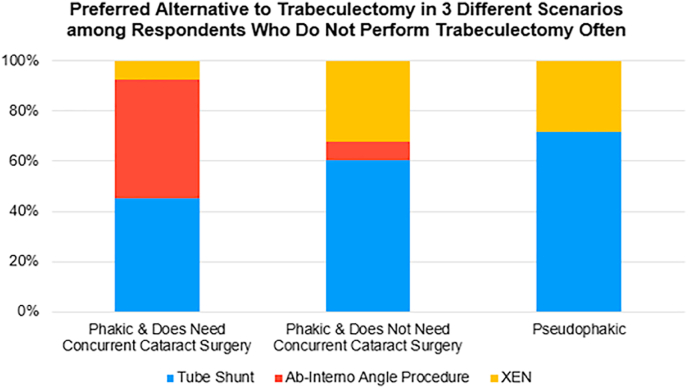
**Self-reported preferred alternatives in the setting of severe-stage POAG among 53 respondents who self-reported performing a low or neutral volume of trabeculectomies in attending practice.** For patients with severe-stage POAG, the most commonly preferred alternative to trabeculectomy depended on the lens status and need for concurrent cataract surgery or lack thereof.

Participants who reported low or neutral self-perceived trabeculectomy volume as an attending physician received an automatic follow-up question to select their preferred alternative procedure in a variety of hypothetical clinical scenarios for a patient with severe-stage primary open angle glaucoma (POAG) (Fig. 3). If the hypothetical eye requires concurrent cataract surgery, 47.2% of respondents reported preferring an ab-interno angle procedure over trabeculectomy, whereas 45.3% reported preferring a tube shunt over trabeculectomy. If the hypothetical eye was phakic without a visually significant cataract, 60.4% reported preferring a tube shunt over trabeculectomy. If the hypothetical eye was pseudophakic, 71.7% reported preferring a tube shunt over trabeculectomy (Fig. 3).

**Fig. 4:**
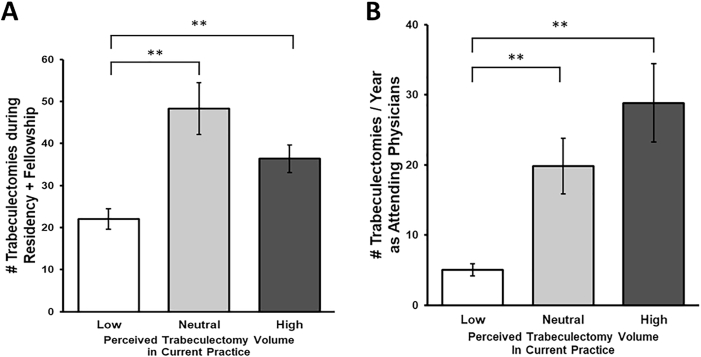
**Self-reported trabeculectomy volume during training and as attending physicians, stratified by self-reported perception of trabeculectomy volume as attending physicians.** (A) Self-reported trabeculectomy volume during training was significantly associated with self-reported trabeculectomy volume in attending practice. (B) Respondents who self-reported low trabeculectomy volume in current attending practice did report lower estimated trabeculectomy volume in attending practice than those who self-reported high trabeculectomy use during attending practice. Respondents' perceived trabeculectomy volume aligns with their reported volume as attending physicians. ∗∗*P* ​< ​0.01.

Participants were asked to report the number of trabeculectomies they had performed in both residency and fellowship as well as provide an estimate of how many trabeculectomies they typically perform annually as an attending (Fig. 4). The mean number of trabeculectomies performed during training among those who self-report performing a high volume of trabeculectomies in their current practice (N ​= ​13) was 36.4 trabeculectomies during training, compared to 22.0 trabeculectomies among those who report a low volume of trabeculectomies in their current practice (N ​= ​42) (*P* ​< ​0.001). The difference in number of trabeculectomies performed as attendings between these two groups mirrored their self-reported statuses, with the high trabeculectomy group performing an average of 28.8 trabeculectomies per year compared to 5.0 trabeculectomies per year in the low trabeculectomy group (*P* ​< ​0.001).

**Fig. 5:**
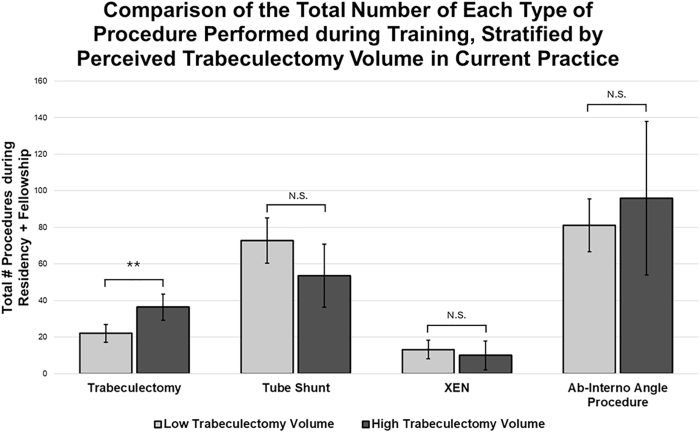
**Comparison of the mean total procedures performed as trainees, stratified by low (N ​= ​42) and high (N ​= ​13) perceived trabeculectomy volume in current practice.** Those who report performing trabeculectomy infrequently as attendings trended towards a higher number of tubes, XEN Gel Stents, and ab-interno angle procedures during training compared to those who report performing trabeculectomy frequently as attendings. While none of these comparisons reached statistical significance, besides the trabeculectomy group, there is a trend of increasing volume procedure numbers in the Low Trabeculectomy Volume group. ∗∗*P* ​< ​0.01; N.S. No Significance.

Fig. 5 compares the total number of each procedure performed during training in the cohort of respondents who reported a low versus high volume of trabeculectomies in their current practice. While there were no statistically significant differences in the volume of tube shunt (*P* ​= ​0.11), XEN Gel Stent (*P* ​= ​0.53), or ab-interno angle procedures (*P* ​= ​0.38) performed by these two groups, those who reported performing trabeculectomy infrequently (N ​= ​42) trended toward a higher median number of tube shunt (low: 62, high: 50), XEN Gel Stent (low: 5.5, high: 1), and ab-interno procedures (low: 77.5, high: 60) completed as residents and fellows compared to those who report a high volume of trabeculectomy (N ​= ​13).

Those who reported performing trabeculectomy often (N ​= ​13) were most influenced by the high success rates of trabeculectomy (N ​= ​12, 92.3%), low instances of postoperative complications (N ​= ​8, 61.5%), and the surgery's preoperative (N ​= ​10, 76.9%) and intraoperative (N ​= ​11, 84.6%) processes (Supplementary Fig. 1A). Those who reported not performing trabeculectomy often (N ​= ​42) were most influenced by the procedure's post-operative process (69.0%) and the socioeconomic (66.7%) and sociocultural (52.4%) characteristics of their patient. Other factors such as volume performed in training (47.6%), cost-effectiveness (19.0%), and reimbursement (19.0%), were reported to be less notable contributors (Supplementary Fig. 1A). When offered the option to provide additional commentary regarding why they do or do not frequently perform trabeculectomies in their current practice, the most common themes mentioned by respondents included considerations for the type of glaucoma being treated, access to appropriate equipment, and the number of patients being treated by the provider. Full free text responses provided by participants can be found in Supplemental Digital Content 2.

The same questions regarding possible factors that influence procedure choice were repeated for tube shunts, XEN Gel Stent, and ab-interno angle procedures. Of the 28 respondents who reported feeling that they perform tube shunt procedures often (42.4%), 22 respondents (78.6%) agreed that the high volume they performed in training contributed to their frequent use of tube shunts as attendings (Supplementary Fig. 1B). Other factors that were reported to influence performing tube shunts often included both the intra-operative and post-operative processes (82.1%). Among the 14 respondents (21.2%) who reported that they perform tube shunts infrequently, only 1 individual (7.1%) reported that they feel that the low volume of tube shunts that they performed during training contributes to why they do not perform tube shunts often as an attending (Supplementary Fig. 1B).

When asked about their perceived use of XEN Gel Stent procedures (ab-externo or ab-interno) in their current attending practice, only 7 of the 66 total participants (10.6%) reported that they perform XENs often (Fig. 2). Among this group, factors that contributed to why they perform XEN often included the post-operative process (85.7%), operative time (71.4%) and technical difficulty (71.4%) relative to other glaucoma surgeries often (Supplementary Fig. 1C). Among the 50 (75.8%) participants that reported that they do not perform XEN often, reported factors included the XEN failure rates observed during training (74.0%) and the wound healing characteristics of the patients that they serve as an attending (54.0%) often (Supplementary Fig. 1C).

When asked about their perceived use of ab-interno angle procedures in their current attending practice, 50 of the 66 respondents (75.8%) reported that they perform them often, whereas only 4 respondents (6.1%) reported that they do not perform ab-interno angle procedures often (Supplementary Fig. 1D). Among those that report frequently performing ab-interno angle procedures, contributing factors included the intra- and post-operative processes (88.0%), low early and late post-operative complications (86.0%), and the operative time (84.0%). While 76.0% of those who self-report frequently performing ab-interno angle surgeries agreed that they are influenced in part by the high volume of ab-interno angle procedures that they performed during training, none of the 4 individuals who self-reported that they do not perform ab-interno angle procedures often attributed this to performing a low volume of ab-interno angle procedures during training.

Lastly, only 19 of 66 respondents (28.8%) somewhat or strongly agreed with the notion that all graduating ophthalmology residents should be required to perform a minimum number of trabeculectomies as primary surgeon during their residency training. However, 61 of the 66 respondents (92.4%) somewhat or strongly agreed that glaucoma-fellowship trained surgeons (as opposed to comprehensive ophthalmologists) should be expected to feel comfortable performing trabeculectomy.

## 4 Discussion

While trabeculectomy has historically been considered the gold standard IOP-lowering surgery for severe or recalcitrant glaucoma, other alternatives including specific tube shunts, the XEN Gel Stent, and angle-based MIGS, have become increasingly popular among glaucoma specialists in recent years.^4^^,^^5^^,^^17^ The results of our study reflect this trend among young glaucoma specialists who completed fellowship training in the United States within the past 5 years (2018–2022). Notably, although most respondents report performing enough trabeculectomies during training to feel comfortable as an attending physician, they still tend to prefer alternative procedures in their current practice. This result aligns with a recent study showing that glaucoma fellows are performing increasingly more tube shunt procedures and MIGS since the mid-2010s, while trabeculectomies has slowly declined year-to-year during the same period with an average decrease of 0.80 in number performed year-to-year between 2014 and 2020.^18^

In October 2023, the Review Committee for Ophthalmology for the Accreditation Council for Graduate Medical Education (ACGME) revised the minimum case requirements, with graduating ophthalmology residents now being required to perform at least five MIGS as *primary* surgeon, while also being required to perform five or more trabeculectomies and/or tube shunt procedures (or revisions) as *primary or assistant* surgeon.^19^ Formerly, residents were required to perform five "filtering/shunting procedures" (or revisions) as primary surgeon, and some but not all MIGS counted in this category, which made it challenging to accurately track how many MIGS versus traditional glaucoma surgeries residents were actually performing.^17^^,^^20^ Now, with MIGS being separated into its own category and with no requirement for any number of trabeculectomies or tube shunts performed as *primary* surgeon, these 2023 updates to the ophthalmology residency requirements coincide with the sentiments of our young glaucoma specialist survey respondents. The majority of respondents reported feeling that graduating ophthalmology residents should not be required to perform any minimum number of trabeculectomies but graduating glaucoma fellows should be expected to be comfortable with trabeculectomy.

Our respondents were categorized into "high", "neutral" and "low" groups for four common procedures used to treat primary open-angle glaucoma based on self-perceived procedural volume rather than numbers of each cases performed of each procedure type. In order to capture this subjective response, our internally constructed instrument asked each participant to consider their volume for each procedure in relation to other available treatment options.

Among young glaucoma specialists who self-report not performing many trabeculectomies in their attending practice (N ​= ​42), the volume of trabeculectomies they performed during residency and fellowship was not self-reported to be a key contributing factor. This contrasts with the opinion published in an editorial by senior glaucoma specialists that the increase in tube shunt procedures and MIGS being performed during residency and fellowship has led to a decline in experience performing trabeculectomies during training and a corresponding reduction in those being performed in attending practice.^11^ Our results reveal that while many young glaucoma specialists self-report that they do feel adequately trained to perform trabeculectomy when indicated, they often choose alternative procedures instead for a multitude of other reasons.

The degree of comfort with performing trabeculectomies among our respondents following their completion of fellowship was elicited subjectively in order to assess whether or not the respondent feels as though their exposure to the procedure during training was adequate in providing the skills necessary to perform it in practice at attending physicians. Future investigations may benefit from determining a more objective method of obtaining the respondent's self-perception of their own surgical capabilities.

Of the 42 respondents who self-reported that they do not perform many trabeculectomies in their current attending practice, 52.4% did not perceive that the volume of trabeculectomies that they performed during training was a factor that considerably influenced their practice pattern. Nonetheless, there was a statistically significant difference in the total number of trabeculectomies performed during residency and fellowship between the "high" and "low" trabeculectomy groups of respondents (Fig. 4). Within this cohort, there is a correlation between the number of trabeculectomies performed during residency and fellowship training and the volume of trabeculectomies performed in attending practice, despite the contradictory finding that the respondents themselves do not perceive their surgical volumes during training to be the primary factor impacting their procedure choices as an attending physician. This may suggest that individuals might not be fully self-aware of or willing to admit to, even anonymously, the factors that influence their practice patterns. Alternatively, the sample size of the "high trabeculectomy" group may be too small (N ​= ​13) to draw generalizable conclusions, necessitating more power to elucidate this trend.

The preference toward trabeculectomy alternatives in attending practice despite perceived adequate trabeculectomy training suggests that the trend may be driven by factors other than trabeculectomy volume during training, including both clinical and social factors of the patient population. For example, one factor cited by respondents was the post-operative care that is required for trabeculectomies, because many of their patients would likely encounter obstacles that prevent them from adhering to the follow-up regimen. Although there can be similar rates of postoperative complications between trabeculectomy and tube shunts, data has shown that achieving optimal outcomes after trabeculectomy requires more extensive follow-up care and patient adherence.^21^^,^^22^ With increasing evidence that tube shunts can achieve similar IOP outcomes as trabeculectomy in patients with uncontrolled glaucoma at 5 years,^23^ and MIGS offering a less invasive and safer first-line surgical alternative for patients with mild-to-moderate stage disease,^8^^,^^24^ it is not surprising that many young glaucoma specialists may consider these alternatives to trabeculectomy, especially in patients for whom extensive post-operative follow-up care is not possible or practical. Meanwhile, an argument can be made that a lack of attention toward the practice of trabeculectomy can limit the ability to address cases with very low target IOP and intolerance to medication, as discussed previously.^14^ Overall, the implications of this shift away from trabeculectomy in favor of less invasive procedures will be more fully elucidated in years to come.

Furthermore, more than half of the participants who self-report that they do not perform trabeculectomy often agreed that sociocultural background (52.4%) and socioeconomic status (SES) (66.7%) were factors influencing their practice pattern. This supports the literature that non-clinical traits (for example race, smoking status) are also associated with risk of trabeculectomy failure.^25^^,^^26^ Previous studies have yet to find a strong correlation between SES and the effectiveness of different surgical treatments in treating advanced-stage glaucoma.^27^ Nonetheless, socioeconomic factors such as income, insurance eligibility, and access to resources (e.g. transportation, technology, etc.) have been shown to influence a patient's ability to maintain adequate follow-up care to allow for optimal treatment outcomes following glaucoma treatment.^28^ Therefore, the extensive post-operative process of trabeculectomy versus other glaucoma treatment options might pose greater harm to those without the means to routinely follow up with their glaucoma surgeon, dissuading the physician from performing this procedure in patients with risk factors for unreliable postoperative follow-up.

Among respondents who self-report that they perform trabeculectomy often (N ​= ​13), the most cited reason for their perceived high trabeculectomy volume in practice was the high success rate they witnessed during training (92.3%). Conversely, among respondents who self-report that they do not perform trabeculectomy often (N ​= ​42), only 28.6% attribute their perceived low volume of trabeculectomy in practice to witnessing a high failure rate during training. This supports the widely accepted notion that trabeculectomy is an effective IOP-lowering surgery,^29^ and young glaucoma specialists that choose not to perform many trabeculectomies are not being deterred primarily due to concern for surgical failure. These results indicate that our cohort of young specialists understand the utility of trabeculectomy and feel comfortable performing the procedure, suggesting that their decision to often choose alternative glaucoma surgeries in their current practice are largely influenced by other factors.

Complication rates associated with trabeculectomy versus tube shunts, XEN Gel Stent procedures, and MIGS do seem to influence those respondents who do not perform trabeculectomy often. Early post-operative complications in the days to weeks following a trabeculectomy was cited by 57.1% of respondents who do not perform trabeculectomy often as a reason for preferring alternative procedures. Late complications, occurring in the months-to-years following the procedure was also a notable influence on their procedure choice (40.5%). Trabeculectomy has been shown to have a higher complication rate compared to non-penetrating glaucoma surgeries.^4^ While MIGS may not reduce IOP to the extent of a successful trabeculectomy, the safety profile of MIGS seems to be a contributing factor for why these less invasive alternative procedures are increasingly preferred by young glaucoma specialists.^30^ Another reason for choosing MIGS over trabeculectomy is that MIGS can be used for patients in the earlier stages of disease, whereas trabeculectomy is typically reserved for severe or refractory cases.^31^

The preferred alternative procedure among respondents who did not report frequent use of trabeculectomy in current practice depended on the given clinical scenario. There is evidence suggesting that combining cataract surgery with trabeculectomy can result in worse IOP outcomes than trabeculectomy-alone,^32^ which may explain why this cohort of young glaucoma specialists might prefer an alternative IOP-lowering surgery like MIGS in patients who also require cataract surgery.^33^^,^^34^

Our study includes several limitations. First, we performed a nonmandatory survey study with a restricted pool of eligible participants. As with many survey studies, including those conducted within the AGS,^35^^,^^36^ the response rate (N ​= ​66) was limited and thus may not be representative of all those who have recently completed glaucoma fellowship. 302 individuals were eligible to complete glaucoma fellowship between 2018 and 2022, but it is unknown how many received the questionnaire distributed through the AGS email listserv. Thus, the gathered results cannot be generalized to all young glaucoma specialists, but rather only those within the sample available to us.

Future studies aiming to increase the response rate and further clarify practice patterns of fellowship graduates could consider including questions in the mandatory fellowship exit survey; this would require approval by the Fellowship Compliance Committee. A similar method was used to determine the volume of trabeculectomies, tube shunt procedures, and MIGS performed by each graduate.^18^ Thus, further inquiry at the time of the fellowship exit survey regarding anticipated procedural decision-making may offer more comprehensive insight into the practice patterns of newly graduated specialists. Of note, neither the Hydrus Microstent (Ivantus, Inc., Irvine, CA, USA) nor XEN Gel Stent were included in the fellowship exit surveys at the time of this study, so the MIGS trends reported in this analysis do not include these two treatment options. Nonetheless, a follow-up questionnaire would still be needed several years after these exit surveys are conducted to assess whether these individuals' actual practice patterns match their anticipated practice patterns at the end of their fellowship training.

As our questionnaire was not mandatory, the results of this study are subject to response bias as there may be a difference in those who received this questionnaire and chose to participate versus those who declined to do so. As such, glaucoma specialists of certain practice settings or geographic locations, for example, may be underrepresented. Additionally, the nature of this retrospective survey study may result in recall bias as participants are asked to reflect on their individual caseloads over the preceding one to five years of practice. The subjective manner in which we sought self-perceived volume for each type of procedure was intended to limit the effect of such bias by allowing respondents to compare their performance of certain procedures in relation to alternative options.

Next, our questionnaire did not inquire about the composition of each respondent's current patient composition, including whether they treat glaucoma exclusively or are part of a comprehensive ophthalmic practice. This factor might have an impact on whether a recent graduate performs one type of IOP-lowering procedure more than another or otherwise affects provider preferences. This would be an interesting area of focus for future investigations.

Furthermore, this study utilized a questionnaire developed by the authors and has not been externally validated. However, to help address this, questions were developed with the input of several glaucoma specialists, and opportunities were offered to provide additional explanation in instances where answer options provided may not be sufficient or applicable. Likewise, the construct validity of our instrument is limited. This can be addressed in future studies through further refinement of the questions based on collected responses and input.

Due to the anonymity of responses, we are unable to compare the characteristics of non-respondents and respondents to our study. Similarly, to maintain respondent anonymity, data regarding age, gender, and race/ethnicity were not collected. As such, we are not able to determine whether specific demographics of young glaucoma surgeons may influence their practice patterns, though this would be an interesting area for future investigation.

Finally, a particularly notable consideration is the timing of survey participants' training with the COVID-19 pandemic. Although not specifically addressed by our study, the pandemic may have conceivably impacted both training experiences (e.g. number of cases) as well as future practice patterns (e.g. practice setting, surgical volume).

## 5 Conclusions

There has been a shift in recent decades towards more tube shunts, XEN Gel Stent procedures, and angle-based MIGS with steady decline in trabeculectomies performed by glaucoma specialists during their training or as practicing attendings.^10^^,^^13^

This trend was reflected in our cohort of recently graduated glaucoma specialists, who tended to choose alternative IOP-lowering surgeries over trabeculectomy depending on the clinical scenario. Our results show that this sample of young glaucoma specialists do not primarily attribute this trend to inadequate trabeculectomy volume during their training. Rather, this sample of young glaucoma specialists report that their tendency toward performing fewer trabeculectomies, despite feeling adequately trained, is influenced by a variety of clinical and non-clinical factors that make alternative IOP-lowering surgeries more suitable. The objective of this study was to collect this data to help inform further investigations into the impact of training experiences and alternative factors on the practice patterns of the newly trained glaucoma surgeons.

Furthermore, the recent changes in ophthalmology residency requirements, which eliminated the requirement to perform any number of trabeculectomies or tube shunts as primary surgeon and included the new requirement to perform at least five MIGS procedures as primary surgeon, suggest that MIGS will still be performed by future generations of comprehensive ophthalmologists. Meanwhile, trabeculectomies and tube shunts may become increasingly reserved for fellowship-trained glaucoma specialists. These updated residency requirements may allow glaucoma specialists to become even more comfortable performing traditional glaucoma surgeries if there is a strong emphasis on trabeculectomies and tube shunts in glaucoma fellowship rather than spreading out this experience across both residency and fellowship. Future longitudinal studies are needed to elucidate the training and clinical decision-making of glaucoma specialists at the time of their fellowship graduation and follow changes in these perceptions over time as respondents begin their clinical practice as attending physicians.

## Study approval

The authors confirm that any aspect of the work covered in this manuscript that involved human patients or animals was conducted with the ethical approval of all relevant bodies and the ​study was performed in accordance with the Declaration of Helsinki.

## Author contributions

The authors confirm contribution to the paper as follows: ​Conception and design of study: NM, MQ; Data collection: NM; Analysis and interpretation of results: NM, JZ, MQ; Drafting the manuscript: SK, WB, JM; ​All authors reviewed the results and approved the final version of the manuscript.

## Funding

This research did not receive any specific grant from funding agencies in the public, commercial, or not-for-profit sectors.

## Declaration of competing interest

The authors declare that they have no known competing financial interests or personal relationships that could have appeared to influence the work reported in this paper.
