# Supplementary material for: Young glaucoma specialist practice patterns: Why do you do what you do?
Source: Adv Ophthalmol Pract Res. 2025 Jul 4;5(4):227–34. doi: 10.1016/j.aopr.2025.07.001 (PMC12684888; doi:10.1016/j.aopr.2025.07.001)
Supplement: Multimedia component 1 [file mmc1.pdf]

Glaucoma Specialist Training and its Influence on Procedure Preference

Please complete the survey below.

Thank you!

What year did you complete glaucoma fellowship training?	◯ 2018<br>◯ 2019<br>◯ 2020<br>◯ 2021<br>◯ 2022
What is your practice setting?	☐ Academic<br>☐ Private practice<br>☐ VA<br>☐ County<br>☐ Other
Please describe the setting in which you primarily practice.	__________________________________
Which of the following best describes the type of region you practice in?	☐ Rural<br>☐ Urban<br>☐ Suburban<br>☐ Other
Please describe the region in which you practice	__________________________________
How many residents per class were in your residency program?	____________________________
How many fellows per class were in your fellowship program?	____________________________

Trabeculectomies

How many trabeculectomies did you perform as primary surgeon during residency? __________________________________

How many trabeculectomies did you perform as primary surgeon during fellowship? __________________________________

Approximately how many trabeculectomies do you typically perform as an attending physician in a given month? __________________________________

Approximately how many trabeculectomies do you perform as an attending physician in a typical year? __________________________________

Do you perceive that you do a lot of trabeculectomies in your current practice as a glaucoma specialist?

(we understand that perceptions of case volumes vary from surgeon to surgeon and what may be a lot to one may not be a lot to another- please answer this question based on how you feel about the number/volume of trabeculectomies you perform)

- ◯ Yes
- ◯ No
- ◯ Neutral

	Tube shunt	XEN (ab-interno or ab-externo)	Ab-interno angle procedure
Which procedure do you most often choose instead of a trabeculectomy for severe-stage POAG if the patient also needs concurrent cataract surgery	◯	◯	◯
Which procedure do you most often choose instead of a trabeculectomy for severe-stage POAG if the patient is phakic and does not need concurrent cataract surgery	◯	◯	◯
Which procedure do you most often choose instead of a trabeculectomy for severe-stage POAG if the patient is already psuedophakic	◯	◯	◯

Do you feel comfortable performing trabeculectomies in your current practice as a result of your training?

- ◯ Yes
- ◯ No
- ◯ Somewhat

Which of the following statements best describes how you feel about your current practice as a glaucoma specialist:	◯ I feel comfortable performing trabeculectomies as a result of my training, and thus I choose to perform trabeculectomies often in my current patient population<br>◯ Despite feeling comfortable performing trabeculectomies as a result of my training, I do not perform trabeculectomies often because I do not feel that it is appropriate for my current patient population
Are you currently or have you in the past sought out additional training/instruction as an attending physician to help you improve your ability to perform trabeculectomies?	◯ Yes<br>◯ No
Please describe any type of additional resources/instruction you have sought out to learn how to improve your ability to perform a trabeculectomy	______________________________
Do you believe that having access to a surgical mentor through the American Glaucoma Society could help improve your comfort level with performing trabeculectomies and benefit your current practice?	◯ Yes<br>◯ No

To what extent do you agree with the following statements regarding how these factors influence your practice patterns when treating patients with glaucoma?

	Strongly disagree	Somewhat disagree	Neither agree nor disagree	Somewhat agree	Strongly agree
I perform a high volume of trabeculectomies now as an attending physician because I performed a high volume of trabeculectomies during my training	◯	◯	◯	◯	◯
I perform a high volume of trabeculectomies now as an attending physician because of the high success rates I saw during my training in patients who received this procedure	◯	◯	◯	◯	◯
I perform a high volume of trabeculectomies now as an attending physician because of the low rate of early post-op complications (i.e.in the days to weeks following surgery) that I saw during my training in patients who received this procedure	◯	◯	◯	◯	◯
I perform a high volume of trabeculectomies now as an attending physician because of the low rate of late post-op complications (i.e.in the months to years following surgery) that I saw during my training in patients who received this procedure	◯	◯	◯	◯	◯
My experiences and observations of the pre-operative treatment planning process for patients during my training influences how many trabeculectomies I perform as an attending physician	◯	◯	◯	◯	◯

My experiences and observations of the intra-operative process of performing trabeculectomies during my training encourages me to perform more trabeculectomies today as an attending physician	○	○	○	○	○
The post-operative care process required for patients who receive a trabeculectomy encourages me to perform this procedure more often in my current practice	○	○	○	○	○
The reimbursement rates for performing trabeculectomies encourages me to perform these procedures today as an attending physician	○	○	○	○	○
The overall cost-effectiveness of performing trabeculectomies encourages me to perform these procedures more often	○	○	○	○	○
The technical difficulty of a trabeculectomy compared to alternative procedures encourages me to choose this treatment option for my patients	○	○	○	○	○
The length of time it takes to complete a trabeculectomy encourages me to perform this procedure more often	○	○	○	○	○
The wound healing characteristics of the patients I serve encourages me to perform trabeculectomies more often in my current practice	○	○	○	○	○
Sociocultural features of the patient population I serve (i.e. primary language, race/ethnicity, cultural background) influences how many trabeculectomies I perform today as an attending physician	○	○	○	○	○

Socioeconomic features of the patient population I serve (i.e. access to transportation, primary insurance coverage) influences how many trabeculectomies I perform today as an attending physician	◯	◯	◯	◯	◯
I perform a low volume trabeculectomies now as an attending physician because I do not feel as though I performed enough trabeculectomies during my training	◯	◯	◯	◯	◯
I do not perform as many trabeculectomies now as an attending physician because of the high failure rates I saw during my training in patients who received this procedure	◯	◯	◯	◯	◯
I choose not to perform as many trabeculectomies now as an attending physician because of the high rate of early post-op complications (i.e.in the days to weeks following surgery) that I saw during my training in patients who received this procedure	◯	◯	◯	◯	◯
I choose not to perform as many trabeculectomies now as an attending physician because of the high rate of late post-op complications (i.e. in the months to years following surgery) that I saw during my training in patients who received this procedure	◯	◯	◯	◯	◯
My experiences and observations of the pre-operative treatment planning process for patients during my training influences how many trabeculectomies I perform as an attending physician	◯	◯	◯	◯	◯

My experiences and observations of the intra-operative process of performing trabeculectomies during my training discourages me from performing more trabeculectomies today as an attending physician	○	○	○	○	○
The post-operative care process required for patients who receive a trabeculectomy discourages me from performing this procedure more often in my current practice	○	○	○	○	○
The reimbursement rates for performing trabeculectomies discourages me from performing these procedures today as an attending physician	○	○	○	○	○
The overall cost-effectiveness of performing trabeculectomies discourages me from performing these procedures more often	○	○	○	○	○
The technical difficulty of a trabeculectomy compared to alternative procedures discourages me from choosing this treatment option for my patients	○	○	○	○	○
The length of time it takes to complete a trabeculectomy discourages me from performing this procedure more often	○	○	○	○	○
I have difficulty obtaining mitomycin C in my current practice, which discourages me from performing this procedure more often	○	○	○	○	○
I do not perform many trabeculectomies due to difficulties faced in obtaining proper anesthesia care in my current place of practice	○	○	○	○	○

The wound healing characteristics of the patients I serve discourages me from performing trabeculectomies more often in my current practice	○	○	○	○	○
Sociocultural features of the patient population I serve (i.e. primary language, race/ethnicity, cultural background) influences how many trabeculectomies I perform today as an attending physician	○	○	○	○	○
Socioeconomic features of the patient population I serve (i.e. access to transportation, primary insurance coverage) influences how many trabeculectomies I perform today as an attending physician	○	○	○	○	○
The volume of trabeculectomies I performed during my training impacts the volume of trabeculectomies I perform now as an attending physician	○	○	○	○	○
The success vs failure rate of trabeculectomies that I saw during my training has influenced the volume of trabeculectomies I perform now as an attending physician	○	○	○	○	○
The rate of early post-op complications (i.e.in the days to weeks following surgery) that I saw during my training in patients who received this procedure has influenced the volume of trabeculectomies I perform now as an attending physician	○	○	○	○	○

The rate of late post-op complications (i.e. in the months to years following surgery) that I saw during my training in patients who received this procedure has influenced the volume of trabeculectomies I perform now as an attending physician	○	○	○	○	○
My experiences and observations of the pre-operative treatment planning process for patients during my training influences how many trabeculectomies I perform as an attending physician	○	○	○	○	○
My experiences and observations of the intra-operative process of performing trabeculectomies during my training influences how many trabeculectomies I perform as an attending physician	○	○	○	○	○
The post-operative care process required for patients who receive a trabeculectomy influences how many trabeculectomies I perform today as an attending physician	○	○	○	○	○
The reimbursement rates for performing trabeculectomies influences how many trabeculectomies I perform today as an attending physician	○	○	○	○	○
The overall cost-effectiveness of performing trabeculectomies influences how many trabeculectomies I perform today as an attending physician	○	○	○	○	○
The technical difficulty of a trabeculectomy compared to alternative procedures influences how many trabeculectomies I perform today as an attending physician	○	○	○	○	○

The length of time it takes to complete a trabeculectomy influences how many trabeculectomies I perform today as an attending physician	○	○	○	○	○
The process of obtaining mitomycin C in my current practice influences how many trabeculectomies I perform today as an attending physician	○	○	○	○	○
The wound healing characteristics of the patients I serve influences how many trabeculectomies I perform today as an attending physician	○	○	○	○	○
Sociocultural features of the patient population I serve (i.e. primary language, race/ethnicity, cultural background) influences how many trabeculectomies I perform today as an attending physician	○	○	○	○	○
Socioeconomic features of the patient population I serve (i.e. access to transportation, primary insurance coverage) influences how many trabeculectomies I perform today as an attending physician	○	○	○	○	○

To what extent do you agree with the following statements regarding ophthalmology training and trabeculectomies?

	Strongly disagree	Somewhat disagree	Neither agree nor disagree	Somewhat agree	Strongly agree
I believe that graduating ophthalmology residents should be required to perform a minimum number of trabeculectomies as primary surgeon?	○	○	○	○	○
I believe that only glaucoma fellowship-trained surgeons should be expected to feel comfortable performing trabeculectomies (i.e., we should not expect comprehensive ophthalmologists who did not complete glaucoma fellowship training to feel comfortable performing this procedure)	○	○	○	○	○

How many trabeculectomies do you believe a trainee would need to perform as primary surgeon during their training (residency and fellowship combined) in order to feel comfortable performing this procedure as an attending physician? __________________________________

Please describe any additional factors that influence how many trabeculectomies you do in your current practice: ____

Tube Shunt Procedures

How many tube shunt procedures did you perform as primary surgeon during residency? __________________________________

How many tube shunt procedures did you perform as primary surgeon during fellowship? __________________________________

Approximately how many tube shunt procedures do you typically perform as an attending physician in a given month? __________________________________

Approximately how many tube shunt procedures do you perform as an attending physician in a typical year? __________________________________

Do you perceive that you do a lot of tube shunt procedures in your current practice as a glaucoma specialist?

(we understand that perceptions of case volumes vary from surgeon to surgeon and what may be a lot to one may not be a lot to another- please answer this question based on how you feel about the number/volume of tube shunt procedures you perform)

- ◯ Yes
- ◯ No
- ◯ Neutral

	Trabeculectomy	XEN (ab-interno or ab-externo)	Ab-interno angle procedure
Which procedure do you most often choose instead of a tube shunt procedure for severe-stage POAG if the patient needs concurrent cataract surgery	◯	◯	◯
Which procedure do you most often choose instead of a tube shunt procedure for severe-stage POAG if the patient is phakic and does not need concurrent cataract surgery	◯	◯	◯
Which procedure do you most often choose instead of a tube shunt procedure for severe-stage POAG if the patient is already pseudophakic	◯	◯	◯

Do you feel comfortable performing tube shunt procedures as a result of your training?

- ◯ Yes
- ◯ No
- ◯ Somewhat

Which of the following statements best describes how you feel about your current practice as a glaucoma specialist:	◯ I feel comfortable performing tube shunt procedures as a result of my training, and thus I choose to perform this procedure often in my current patient population<br>◯ Despite feeling comfortable performing tube shunt procedures as a result of my training, I do not perform this procedure often because I do not feel that it is appropriate for my current patient population
Are you currently or have you in the past sought out additional training/instruction as an attending physician to help you improve your ability to perform tube shunt procedures?	◯ Yes<br>◯ No
Please describe any type of additional resources/instruction you have sought out to learn how to improve your ability to perform a tube shunt procedure	__________________________________
Do you believe that having access to a surgical mentor through the American Glaucoma Society could help improve your comfort level with performing tube shunt procedures and benefit your current practice?	◯ Yes<br>◯ No

To what extent do you agree with the following statements regarding how these factors influence your practice patterns when treating patients with glaucoma?

	Strongly disagree	Somewhat disagree	Neither agree nor disagree	Somewhat agree	Strongly agree
I perform a high volume of tube shunt procedures now as an attending physician because I performed a high volume of tube shunt procedures during my training	○	○	○	○	○
I perform a high volume of tube shunt procedures now as an attending physician because of the high success rates I saw during my training in patients who received this procedure	○	○	○	○	○
I perform a high volume of tube shunt procedures now as an attending physician because of the low rate of early post-op complications (i.e.in the days to weeks following surgery) that I saw during my training in patients who received this procedure	○	○	○	○	○
I perform a high volume of tube shunt procedures now as an attending physician because of the low rate of late post-op complications (i.e.in the months to years following surgery) that I saw during my training in patients who received this procedure	○	○	○	○	○
My experiences and observations of the pre-operative treatment planning process for patients during my training influences how many tube shunt procedures I perform as an attending physician	○	○	○	○	○

My experiences and observations of the intra-operative process of performing tube shunt procedures during my training encourages me to perform more tube shunt procedures today as an attending physician	○	○	○	○	○
The post-operative care process required for patients who receive a tube shunt procedure encourages me to perform this procedure more often in my current practice	○	○	○	○	○
The reimbursement rates for performing tube shunt procedures encourages me to perform these procedures today as an attending physician	○	○	○	○	○
The overall cost-effectiveness of performing tube shunt procedures encourages me to perform these procedures more often	○	○	○	○	○
The technical difficulty of a tube shunt procedure compared to alternative procedures encourages me to choose this treatment option for my patients	○	○	○	○	○
The length of time it takes to complete a tube shunt procedure encourages me to perform this procedure more often	○	○	○	○	○
The wound healing characteristics of the patients I serve encourages me to perform tube shunt procedures more often in my current practice	○	○	○	○	○
Sociocultural features of the patient population I serve (i.e. primary language, race/ethnicity, cultural background) influences how many tube shunt procedures I perform today as an attending physician	○	○	○	○	○

Socioeconomic features of the patient population I serve (i.e. access to transportation, primary insurance coverage) influences how many tube shunt procedures I perform today as an attending physician	○	○	○	○	○
I perform a low volume tube shunt procedures now as an attending physician because I do not feel as though I performed enough tube shunt procedures during my training	○	○	○	○	○
I do not perform as many tube shunt procedures now an attending physician because of the high failure rates I saw during my training in patients who received this procedure	○	○	○	○	○
I choose not to perform as many tube shunt procedures now as an attending physician because of the high rate of early post-op complications (i.e.in the days to weeks following surgery) that I saw during my training in patients who received this procedure	○	○	○	○	○
I choose not to perform as many tube shunt procedures now as an attending physician because of the high rate of late post-op complications (i.e. in the months to years following surgery) that I saw during my training in patients who received this procedure	○	○	○	○	○
My experiences and observations of the pre-operative treatment planning process for patients during my training influences how many tube shunt procedures I perform as an attending physician	○	○	○	○	○

My experiences and observations of the intra-operative process of performing tube shunt procedures during my training discourages me from performing more tube shunt procedures today as an attending physician	○	○	○	○	○
The post-operative care process required for patients who receive a tube shunt procedure discourages me from performing this procedure more often in my current practice	○	○	○	○	○
The reimbursement rates for performing tube shunt procedures discourages me from performing these procedures today as an attending physician	○	○	○	○	○
The overall cost-effectiveness of performing tube shunt procedures discourages me from performing these procedures more often	○	○	○	○	○
The technical difficulty of a tube shunt procedure compared to alternative procedures discourages me from choosing this treatment option for my patients	○	○	○	○	○
The length of time it takes to complete a tube shunt procedure discourages me from performing this procedure more often	○	○	○	○	○
I do not perform many tube shunt procedures due to difficulties faced in obtaining proper anesthesia care in my current place of practice	○	○	○	○	○
The wound healing characteristics of the patients I serve discourages me from performing tube shunt procedures more often in my current practice	○	○	○	○	○

Sociocultural features of the patient population I serve (i.e. primary language, race/ethnicity, cultural background) influences how many tube shunt procedures I perform today as an attending physician	○	○	○	○	○
Socioeconomic features of the patient population I serve (i.e. access to transportation, primary insurance coverage) influences how many tube shunt procedures I perform today as an attending physician	○	○	○	○	○
The volume of tube shunt procedures I performed during my training impacts the volume of tube shunt procedures I perform now as an attending physician	○	○	○	○	○
The success vs failure rate of tube shunt procedures that I saw during my training has influenced the volume of tube shunt procedures I perform now as an attending physician	○	○	○	○	○
The rate of early post-op complications (i.e.in the days to weeks following surgery) that I saw during my training in patients who received this procedure has influenced the volume of tube shunt procedures I perform now as an attending physician	○	○	○	○	○
The rate of late post-op complications (i.e. in the months to years following surgery) that I saw during my training in patients who received this procedure has influenced the volume of tube shunt procedures I perform now as an attending physician	○	○	○	○	○

My experiences and observations of the pre-operative treatment planning process for patients during my training influences how many tube shunt procedures I perform as an attending physician	○	○	○	○	○
My experiences and observations of the intra-operative process of performing tube shunt procedures during my training influences how many tube shunt procedures I perform as an attending physician	○	○	○	○	○
The post-operative care process required for patients who receive a tube shunt procedure influences how many tube shunt procedures I perform today as an attending physician	○	○	○	○	○
The reimbursement rates for performing tube shunt procedures influences how many tube shunt procedures I perform today as an attending physician	○	○	○	○	○
The overall cost-effectiveness of performing tube shunt procedures influences how many tube shunt procedures I perform today as an attending physician	○	○	○	○	○
The technical difficulty of a tube shunt procedure compared to alternative procedures influences how many tube shunt procedures I perform today as an attending physician	○	○	○	○	○
The length of time it takes to complete a tube shunt procedure influences how many tube shunt procedures I perform today as an attending physician	○	○	○	○	○
The wound healing characteristics of the patients I serve influences how many tube shunt procedures I perform today as an attending physician	○	○	○	○	○

Sociocultural features of the patient population I serve (i.e. primary language, race/ethnicity, cultural background) influences how many tube shunt procedures I perform today as an attending physician	○	○	○	○	○
Socioeconomic features of the patient population I serve (i.e. access to transportation, primary insurance coverage) influences how many tube shunt procedures I perform today as an attending physician	○	○	○	○	○

How many tube shunt procedures do you believe a trainee would need to perform as primary surgeon during their training (residency and fellowship combined) in order to feel comfortable performing this procedure as an attending physician? __________________________________

Please describe any other factors that you find influence the number of tube shunt procedures you tend to perform in your current practice: ____

XEN Procedures

How many XEN procedures did you perform as primary surgeon during residency? __________________________________

How many XEN procedures did you perform as primary surgeon during fellowship? __________________________________

Approximately how many XEN procedures do you typically perform as an attending physician in a given month? __________________________________

Approximately how many XEN procedures do you perform as an attending physician in a typical year? __________________________________

Do you perceive that you do a lot of XEN procedures (ab-externo or ab-interno) in your current practice as a glaucoma specialist?

(we understand that perceptions of case volumes vary from surgeon to surgeon and what may be a lot to one may not be a lot to another- please answer this question based on how you feel about the number/volume of XEN procedures you perform)

◯ Yes
◯ No
◯ Neutral

	Trabeculectomy	Tube shunt	Ab-interno angle procedure
Which procedure do you most often choose instead of a XEN (ab-externo or ab-interno) for severe-stage POAG if the patient needs concurrent cataract surgery	◯	◯	◯
Which procedure do you most often choose instead of a XEN (ab-externo or ab-interno) for severe-stage POAG if the patient is phakic and does not need concurrent cataract surgery	◯	◯	◯
Which procedure do you most often choose instead of a XEN (ab-externo or ab-interno) for severe-stage POAG if the patient is already pseudophakic	◯	◯	◯

Do you feel comfortable performing XEN procedures as a result of your training?

◯ Yes
◯ No
◯ Somewhat

Which of the following statements best describes how you feel about your current practice as a glaucoma specialist:	◯ I feel comfortable performing XEN procedures as a result of my training, and thus I choose to perform this procedure often in my current patient population<br>◯ Despite feeling comfortable performing XEN procedures as a result of my training, I do not perform this procedure often because I do not feel that it is appropriate for my current patient population
Are you currently or have you in the past sought out additional training/instruction as an attending physician to help you improve your ability to perform XEN procedures?	◯ Yes<br>◯ No
Please describe any type of additional resources/instruction you have sought out to learn how to improve your ability to perform a XEN procedure	__________________________________
Do you believe that having access to a surgical mentor through the American Glaucoma Society could help improve your comfort level with performing XEN procedures and benefit your current practice?	◯ Yes<br>◯ No

To what extent do you agree with the following statements regarding how these factors influence your practice patterns when treating patients with glaucoma?

	Strongly disagree	Somewhat disagree	Neither agree nor disagree	Somewhat agree	Strongly agree
I perform a high volume of XEN procedures now as an attending physician because I performed a high volume of XEN procedures during my training	○	○	○	○	○
I perform a high volume of XEN procedures now as an attending physician because of the high success rates I saw during my training in patients who received this procedure	○	○	○	○	○
I perform a high volume of XEN procedures now as an attending physician because of the low rate of early post-op complications (i.e.in the days to weeks following surgery) that I saw during my training in patients who received this procedure	○	○	○	○	○
I perform a high volume of XEN procedures now as an attending physician because of the low rate of late post-op complications (i.e.in the months to years following surgery) that I saw during my training in patients who received this procedure	○	○	○	○	○
My experiences and observations of the pre-operative treatment planning process for patients during my training influences how many XEN procedures I perform as an attending physician	○	○	○	○	○

My experiences and observations of the intra-operative process of performing XEN procedures during my training encourages me to perform more XEN procedures today as an attending physician	○	○	○	○	○
The post-operative care process required for patients who receive a XEN procedure encourages me to perform this procedure more often in my current practice	○	○	○	○	○
The reimbursement rates for performing XEN procedures encourages me to perform these procedures today as an attending physician	○	○	○	○	○
The overall cost-effectiveness of performing XEN procedures encourages me to perform these procedures more often	○	○	○	○	○
The technical difficulty of a XEN procedure compared to alternative procedures encourages me to choose this treatment option for my patients	○	○	○	○	○
The length of time it takes to complete a XEN procedure encourages me to perform this procedure more often	○	○	○	○	○
The wound healing characteristics of the patients I serve encourages me to perform XEN procedures more often in my current practice	○	○	○	○	○
Sociocultural features of the patient population I serve (i.e. primary language, race/ethnicity, cultural background) influences how many XEN procedures I perform today as an attending physician	○	○	○	○	○

Socioeconomic features of the patient population I serve (i.e. access to transportation, primary insurance coverage) influences how many XEN procedures I perform today as an attending physician	○	○	○	○	○
I perform a low volume XEN procedures now as an attending physician because I do not feel as though I performed enough XEN procedures during my training	○	○	○	○	○
I do not perform as many XEN procedures now as an attending physician because of the high failure rates I saw during my training in patients who received this procedure	○	○	○	○	○
I choose not to perform as many XEN procedures now as an attending physician because of the high rate of early post-op complications (i.e.in the days to weeks following surgery) that I saw during my training in patients who received this procedure	○	○	○	○	○
I choose not to perform as many XEN procedures now as an attending physician because of the high rate of late post-op complications (i.e. in the months to years following surgery) that I saw during my training in patients who received this procedure	○	○	○	○	○
My experiences and observations of the pre-operative treatment planning process for patients during my training influences how many XEN procedures I perform as an attending physician	○	○	○	○	○

My experiences and observations of the intra-operative process of performing XEN procedures during my training discourages me from performing more XEN procedures today as an attending physician	◯	◯	◯	◯	◯
The post-operative care process required for patients who receive a XEN procedure discourages me from performing this procedure more often in my current practice	◯	◯	◯	◯	◯
The reimbursement rates for performing XEN procedures discourages me from performing these procedures today as an attending physician	◯	◯	◯	◯	◯
The overall cost-effectiveness of performing XEN procedures discourages me from performing these procedures more often	◯	◯	◯	◯	◯
The technical difficulty of a XEN procedure compared to alternative procedures discourages me from choosing this treatment option for my patients	◯	◯	◯	◯	◯
The length of time it takes to complete a XEN procedure discourages me from performing this procedure more often	◯	◯	◯	◯	◯
I have difficulty obtaining mitomycin C in my current practice, which discourages me from performing this procedure more often	◯	◯	◯	◯	◯
I do not perform many XEN procedures due to difficulties faced in obtaining proper anesthesia care in my current place of practice	◯	◯	◯	◯	◯

The wound healing characteristics of the patients I serve discourages me from performing XEN procedures more often in my current practice	○	○	○	○	○
Sociocultural features of the patient population I serve (i.e. primary language, race/ethnicity, cultural background) influences how many XEN procedures I perform today as an attending physician	○	○	○	○	○
Socioeconomic features of the patient population I serve (i.e. access to transportation, primary insurance coverage) influences how many XEN procedures I perform today as an attending physician	○	○	○	○	○
The volume of XEN procedures I performed during my training impacts the volume of XEN procedures I perform now as an attending physician	○	○	○	○	○
The success vs failure rate of XEN procedures that I saw during my training has influenced the volume of XEN procedures I perform now as an attending physician	○	○	○	○	○
The rate of early post-op complications (i.e.in the days to weeks following surgery) that I saw during my training in patients who received this procedure has influenced the volume of XEN procedures I perform now as an attending physician	○	○	○	○	○

The rate of late post-op complications (i.e. in the months to years following surgery) that I saw during my training in patients who received this procedure has influenced the volume of XEN procedures I perform now as an attending physician	○	○	○	○	○
My experiences and observations of the pre-operative treatment planning process for patients during my training influences how many XEN procedures I perform as an attending physician	○	○	○	○	○
My experiences and observations of the intra-operative process of performing XEN procedures during my training influences how many XEN procedures I perform as an attending physician	○	○	○	○	○
The post-operative care process required for patients who receive a XEN procedure influences how many XEN procedures I perform today as an attending physician	○	○	○	○	○
The reimbursement rates for performing XEN procedures influences how many XEN procedures I perform today as an attending physician	○	○	○	○	○
The overall cost-effectiveness of performing XEN procedures influences how many XEN procedures I perform today as an attending physician	○	○	○	○	○
The technical difficulty of a XEN procedure compared to alternative procedures influences how many XEN procedures I perform today as an attending physician	○	○	○	○	○

The length of time it takes to complete a XEN procedure influences how many XEN procedures I perform today as an attending physician	◯	◯	◯	◯	◯
The process of obtaining mitomycin C in my current practice influences how many XEN procedures I perform today as an attending physician	◯	◯	◯	◯	◯
The wound healing characteristics of the patients I serve influences how many XEN procedures I perform today as an attending physician	◯	◯	◯	◯	◯
Sociocultural features of the patient population I serve (i.e. primary language, race/ethnicity, cultural background) influences how many XEN procedures I perform today as an attending physician	◯	◯	◯	◯	◯
Socioeconomic features of the patient population I serve (i.e. access to transportation, primary insurance coverage) influences how many XEN procedures I perform today as an attending physician	◯	◯	◯	◯	◯

How many XEN procedures do you believe a trainee would need to perform as primary surgeon during their training (residency and fellowship combined) in order to feel comfortable performing this procedure as an attending physician? __________________________________

Please describe any other factors that you find influence the number of XEN procedures you tend to perform in your current practice: __________________________________

Ab-Interno Angle Procedures

How many ab-interno angle procedures did you perform as primary surgeon during residency? __________________________________

How many ab-interno angle procedures did you perform as primary surgeon during fellowship? __________________________________

Approximately how many ab-interno angle procedures do you typically perform as an attending physician in a given month? __________________________________

Approximately how many ab-interno angle procedures do you perform as an attending physician in a typical year? __________________________________

Do you perceive that you do a lot of ab-interno angle procedures in your current practice as a glaucoma specialist?

(we understand that perceptions of case volumes vary from surgeon to surgeon and what may be a lot to one may not be a lot to another- please answer this question based on how you feel about the number/volume of ab-interno angle procedures you perform)

- ◯ Yes
- ◯ No
- ◯ Neutral

	Trabeculectomy	Tube shunt	XEN (ab-externo or ab-interno)
Which procedure do you most often choose instead of an ab-interno angle procedure for severe-stage POAG if the patient needs concurrent cataract surgery	◯	◯	◯
Which procedure do you most often choose instead of an ab-interno angle procedure for severe-stage POAG if the patient is phakic and does not need concurrent cataract surgery	◯	◯	◯
Which procedure do you most often choose instead of an ab-interno angle procedure for severe-stage POAG if the patient is already pseudophakic	◯	◯	◯

Do you feel comfortable performing ab-interno angle procedures as a result of your training?

- ◯ Yes
- ◯ No
- ◯ Somewhat

Which of the following statements best describes how you feel about your current practice as a glaucoma specialist:	◯ I feel comfortable performing ab-interno angle procedures as a result of my training, and thus I choose to perform this procedure often in my current patient population<br>◯ Despite feeling comfortable performing ab-interno angle procedures as a result of my training, I do not perform this procedure often because I do not feel that it is appropriate for my current patient population
Are you currently or have you in the past sought out additional training/instruction as an attending physician to help you improve your ability to perform ab-interno angle procedures?	◯ Yes<br>◯ No
Please describe any type of additional resources/instruction you have sought out to learn how to improve your ability to perform an ab-interno angle procedure	◯ Yes<br>◯ No
Do you believe that having access to a surgical mentor through the American Glaucoma Society could help improve your comfort level with performing ab-interno angle procedures and benefit your current practice?	◯ Yes<br>◯ No

To what extent do you agree with the following statements regarding how these factors influence your practice patterns when treating patients with glaucoma?

	Strongly disagree	Somewhat disagree	Neither agree nor disagree	Somewhat agree	Strongly agree
I perform a high volume of ab-interno angle procedures now as an attending physician because I performed a high volume of ab-interno angle procedures during my training	◯	◯	◯	◯	◯
I perform a high volume of ab-interno angle procedures now as an attending physician because of the high success rates I saw during my training in patients who received this procedure	◯	◯	◯	◯	◯
I perform a high volume of ab-interno angle procedures now as an attending physician because of the low rate of early post-op complications (i.e.in the days to weeks following surgery) that I saw during my training in patients who received this procedure	◯	◯	◯	◯	◯
I perform a high volume of ab-interno angle procedures now as an attending physician because of the low rate of late post-op complications (i.e.in the months to years following surgery) that I saw during my training in patients who received this procedure	◯	◯	◯	◯	◯
My experiences and observations of the pre-operative treatment planning process for patients during my training influences how many ab-interno angle procedures I perform as an attending physician	◯	◯	◯	◯	◯

My experiences and observations of the intra-operative process of performing ab-interno angle procedures during my training encourages me to perform more ab-interno angle procedures today as an attending physician	○	○	○	○	○
The post-operative care process required for patients who receive an ab-interno angle procedure encourages me to perform this procedure more often in my current practice	○	○	○	○	○
The reimbursement rates for performing ab-interno angle procedures encourages me to perform these procedures today as an attending physician	○	○	○	○	○
The overall cost-effectiveness of performing ab-interno angle procedures encourages me to perform these procedures more often	○	○	○	○	○
The technical difficulty of an ab-interno angle procedure compared to alternative procedures encourages me to choose this treatment option for my patients	○	○	○	○	○
The length of time it takes to complete an ab-interno angle procedure encourages me to perform this procedure more often	○	○	○	○	○
The wound healing characteristics of the patients I serve encourages me to perform ab-interno angle procedures more often in my current practice	○	○	○	○	○

Sociocultural features of the patient population I serve (i.e. primary language, race/ethnicity, cultural background) influences how many ab-interno angle procedures I perform today as an attending physician	◯	◯	◯	◯	◯
Socioeconomic features of the patient population I serve (i.e. access to transportation, primary insurance coverage) influences how many ab-interno angle procedures I perform today as an attending physician	◯	◯	◯	◯	◯
I perform a low volume ab-interno angle procedures now as an attending physician because I do not feel as though I performed enough ab-interno angle procedures during my training	◯	◯	◯	◯	◯
I do not perform as many ab-interno angle procedures now as an attending physician because of the high failure rates I saw during my training in patients who received this procedure	◯	◯	◯	◯	◯
I choose not to perform as many ab-interno angle procedures now as an attending physician because of the high rate of early post-op complications (i.e.in the days to weeks following surgery) that I saw during my training in patients who received this procedure	◯	◯	◯	◯	◯
I choose not to perform as many ab-interno angle procedures now as an attending physician because of the high rate of late post-op complications (i.e. in the months to years following surgery) that I saw during my training in patients who received this procedure	◯	◯	◯	◯	◯

My experiences and observations of the pre-operative treatment planning process for patients during my training influences how many ab-interno angle procedures I perform as an attending physician	○	○	○	○	○
My experiences and observations of the intra-operative process of performing ab-interno angle procedures during my training discourages me from performing more ab-interno angle procedures today as an attending physician	○	○	○	○	○
The post-operative care process required for patients who receive an ab-interno angle procedure discourages me from performing this procedure more often in my current practice	○	○	○	○	○
The reimbursement rates for performing ab-interno angle procedures discourages me from performing these procedures today as an attending physician	○	○	○	○	○
The overall cost-effectiveness of performing ab-interno angle procedures discourages me from performing these procedures more often	○	○	○	○	○
The technical difficulty of an ab-interno angle procedure compared to alternative procedures discourages me from choosing this treatment option for my patients	○	○	○	○	○
The length of time it takes to complete an ab-interno angle procedure discourages me from performing this procedure more often	○	○	○	○	○

I do not perform many ab-interno angle procedures due to difficulties faced in obtaining proper anesthesia care in my current place of practice	○	○	○	○	○
The wound healing characteristics of the patients I serve discourages me from performing ab-interno angle procedures more often in my current practice	○	○	○	○	○
Sociocultural features of the patient population I serve (i.e. primary language, race/ethnicity, cultural background) influences how many ab-interno angle procedures I perform today as an attending physician	○	○	○	○	○
Socioeconomic features of the patient population I serve (i.e. access to transportation, primary insurance coverage) influences how many ab-interno angle procedures I perform today as an attending physician	○	○	○	○	○
The number of ab-interno angle procedures I performed as primary surgeon during my residency and fellowship training impacts how many ab-interno angle procedures I perform in my current practice	○	○	○	○	○
The success vs failure rate of ab-interno angle procedures that I saw during my training has influenced the volume of ab-interno angle procedures I perform now as an attending physician	○	○	○	○	○

The rate of early post-op complications (i.e.in the days to weeks following surgery) that I saw during my training in patients who received this procedure has influenced the volume of ab-interno angle procedures I perform now as an attending physician	◯	◯	◯	◯	◯
The rate of late post-op complications (i.e. in the months to years following surgery) that I saw during my training in patients who received this procedure has influenced the volume of ab-interno angle procedures I perform now as an attending physician	◯	◯	◯	◯	◯
My experiences and observations of the pre-operative treatment planning process for patients during my training influences how many ab-interno angle procedures I perform as an attending physician	◯	◯	◯	◯	◯
My experiences and observations of the intra-operative process of performing ab-interno angle procedures during my training influences how many ab-interno angle procedures I perform as an attending physician	◯	◯	◯	◯	◯
The post-operative care process required for patients who receive an ab-interno angle procedure influences how many ab-interno angle procedures I perform today as an attending physician	◯	◯	◯	◯	◯
The reimbursement rates for performing ab-interno angle procedures influences how many ab-interno angle procedures I perform today as an attending physician	◯	◯	◯	◯	◯

The overall cost-effectiveness of performing ab-interno angle procedures influences how many ab-interno angle procedures I perform today as an attending physician	○	○	○	○	○
The technical difficulty of an ab-interno angle procedure compared to alternative procedures influences how many ab-interno angle procedures I perform today as an attending physician	○	○	○	○	○
The length of time it takes to complete an ab-interno angle procedure influences how many ab-interno angle procedures I perform today as an attending physician	○	○	○	○	○
The wound healing characteristics of the patients I serve influences how many ab-interno angle procedures I perform today as an attending physician	○	○	○	○	○
Sociocultural features of the patient population I serve (i.e. primary language, race/ethnicity, cultural background) influences how many ab-interno angle procedures I perform today as an attending physician	○	○	○	○	○
Socioeconomic features of the patient population I serve (i.e. access to transportation, primary insurance coverage) influences how many ab-interno angle procedures I perform today as an attending physician	○	○	○	○	○

How many ab-interno angle procedures do you believe a trainee would need to perform as primary surgeon during their training (residency and fellowship combined) in order to feel comfortable performing this procedure as an attending physician? ______________________

Please describe any other factors that you find influence the number of ab-interno angle procedures you tend to perform in your current practice ______________________
