# Supplementary material for: Young glaucoma specialist practice patterns: Why do you do what you do?
Source: Adv Ophthalmol Pract Res. 2025 Jul 4;5(4):227–34. doi: 10.1016/j.aopr.2025.07.001 (PMC12684888; doi:10.1016/j.aopr.2025.07.001)
Supplement: Multimedia component 2 [file mmc2.pdf]

Supplemental content 2. Comments left by respondents when offered the option to describe any additional factors that influence how many trabeculectomies they perform in their current practice.

**Self-Perceived Trabeculectomy Volume is *High***	**Theme**
Stage of disease and failure of past surgeries	Stage of disease and failure of past surgeries
Volume of glaucoma patients	Patient volume
High success rates compared to other techniques	High success rate
Mentors and seeing pre/post operative management is key to feeling confident in performing trabs. If there is a lack of management it is very discouraging to perform the trab to begin with given the many number of potential early changes and complications.	Mentorship and training experience
Demographics and disease profile of patients seen in practice	Patient demographics
It's mostly the high success rate.	High success rate
Glaucoma type/mechanism	Disease profile
I had a very broad glaucoma fellowship - we performed all glaucoma procedures currently available. Therefore, if the patient needs a trab, I do a trab. I do think that other physicians who don't have trab experience in fellowship, or have limited trab experience with bad outcomes, are less likely to perform them.	Mentorship and training experience
**Self-Perceived Trabeculectomy Volume is *Low***	**Theme**
patient's access to follow up care. often patients live far away and difficult to drive to postop apts	Access to post-op care
Trabs are limited in my practice mostly due to patient factors related to follow-up concerns.	Access to post-op care
I think trabeculectomy still has a role in glaucoma care, but I am more selective in who I think a good patient is. There are many patients I prefer to do tube shunts or xens on because I feel these procedures are safer and will be more successful in many cases. I often perform trabeculectomy for NTG, for patients who need to be fully off drops, or for patients for whom I anticipate multiple procedures during the course of their lifetime.	Other procedures safer and more successful
mostly post-operative care	Post-op care
Not having access to LSL equipment.	Access to LSL equipment

less normal tension glaucoma	Less normal tension glaucoma
Expected time to vision recovery, if patients are monocular or will need the second eye done once they see well enough out of the first time. If patients cannot commit to the prolonged post-op course required.	Post-op care
Alternative options with comparable outcomes and less post-op complications	Alternative options with comparable outcomes and fewer complications
I reserve this procedure for patients with severe disease, or moderate disease with a high rate of progression and uncontrolled medically, failed and angle based procedure or need to get off drops due to compliance, ineffectiveness, or intolerance. The amount of patients I have that meet this criteria is minimal (1 every 1-3 weeks) however they have been increasing in this last year due to patients now failing previously preformed MIGS and increasing referrals from community based providers	Reserved for severe disease and uncontrolled medically
Post operative success and predictability of Baerveldt	Post operative success and predictability of Baerveldt
I just don't have that many patients who need single digits	Few patients who need single digits
lack of an argon laser at satellite locations	Access to LSL equipment
post op care and compliance is limiting factor. also patient characteristics	Post-op care
In the mid-south, many of my patients have factors that, IMO, preclude them from getting a trab, including certain glaucoma sub-types (NVG, uveitic glaucoma), >90 min commute (which makes presenting for close f/u and/or acute PO issue following trab difficult), and f/u and Med/compliance issues. I also am reluctant to perform trabs wherein patients have VA <20/200 and/or fixation-splitting defects (as opposed to preserved central island) given risk of addition VA loss from fluctuating IOP postop (based on my observations in practice and training). For me, the ideal trab candidate is a motivated patient with severe CACG/POAG with preserved central vision, a hx of good follow up and Med compliance, without evidence of conj scarring superiorly, who needs IOP <low teens. I've most often utilized trabs for patients who are pan-allergic to glaucoma meds. Since I end up doing so few per year, I'm primary surgeon for all of these cases.	Types of glaucoma, Access to post-op care
Access to argon laser	Access to LSL equipment

My fellowship director didn't do trabs anymore as felt Xen and tubes were safer and worked. Therefore I did not get trab exposure during fellowship so therefore I don't feel comfortable doing the surgery.	Limited training
I work in a tertiary referral center, and often am referred patients that have failed one or more trab already by another surgeon.	Previously failed trab
Types of glaucoma. I reside in a very sick state, at an academic center with high Medicaid volume. A lot of NVG in which I would never consider a trab. Also, took over someone's 20year established clinic, so most of my POAGS have already had a trab, and if they need surgery doing revisions or moving in to tubes.	Types of glaucoma
I perform a very small number of trabs because I work with a predominantly Hispanic patient population that often times have issues with compliance with meds and appointments.	Patient compliance
Difficult access to argon laser for suture lysis	Access to LSL equipment
i was not trained to preform them	Limited training
Many barriers to follow up (transportation, socioeconomic) and medication adherence (co-morbid mental health diagnoses) in VA population where I practice makes me hesitant to pursue this surgical option more often although I strongly believe it remains the gold-standard for long-term IOP reduction and that Xen/MIGS in no way approaches the success rates for these advanced or quickly progressing patients.	Access to post-op care, Patient compliance
As above, transportation is extremely limited making follow-up challenging.	Access to post-op care
**Self-Perceived Trabeculectomy Volume is *Neutral***	**Theme**
I would do more but i'm primarily research and rather low volume surgically overall	Low surgical volume
If patient has a history of inconsistent follow up I choose a tube as an initial surgery instead of a trab. If the patient has some cataract and glaucoma progression is slow I try phaco/hydrus before moving to trab. If a visual field is still full I try to avoid doing a trab unless I have exhausted other options. I strongly believe that in a reliable patient who will follow up trab is the best surgery for long term control of progressing, visually significant glaucoma.	Access to post-op care
OR time availability	OR time availability
Patient expectations for recovery, use of blood thinners	Patient expectations for recovery
Tertiary referral academic center seeing severe stage glaucoma uncontrolled on maximum tolerated medical therapy	Reserved for severe disease and uncontrolled medically
