# Supplementary material for: Young glaucoma specialist practice patterns: Why do you do what you do?
Source: Adv Ophthalmol Pract Res. 2025 Jul 4;5(4):227–34. doi: 10.1016/j.aopr.2025.07.001 (PMC12684888; doi:10.1016/j.aopr.2025.07.001)

**Supplementary Figure 1**. **Factors influencing procedure preference.** **A-D,** Most common factors that influenced procedure preference among those who self-reported performing a low and high volume of (A) trabeculectomies, (B) tube shunts, (C) XEN Gel Stents, and (D) ab-interno angle procedures.

1. **Trabeculectomy**


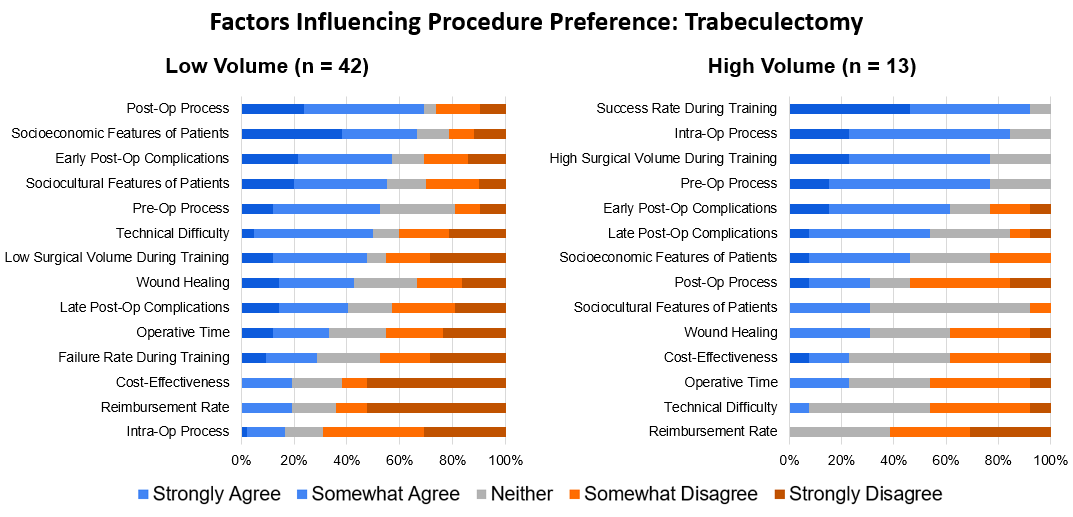


**(B) Tube Shunt**


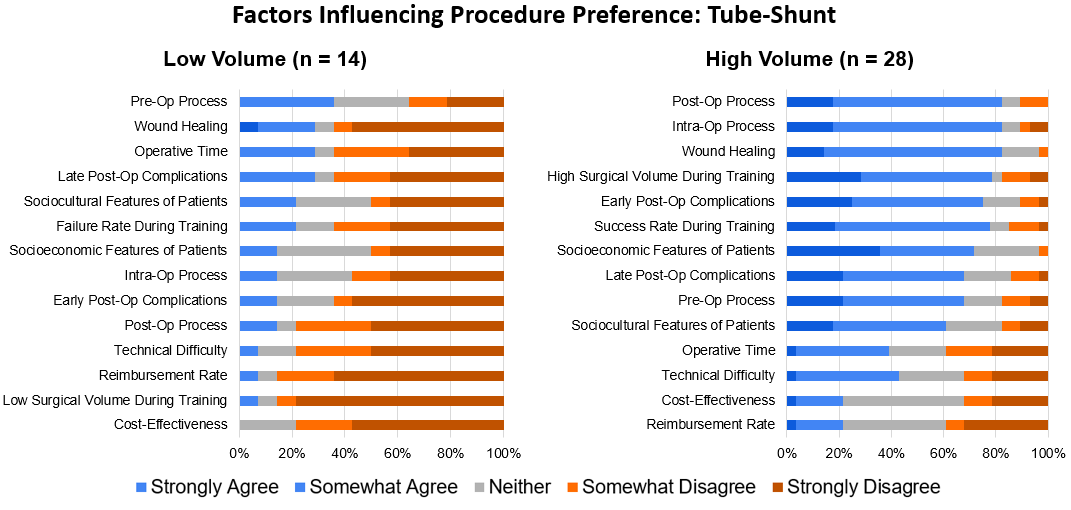


**(C) XEN Procedure**


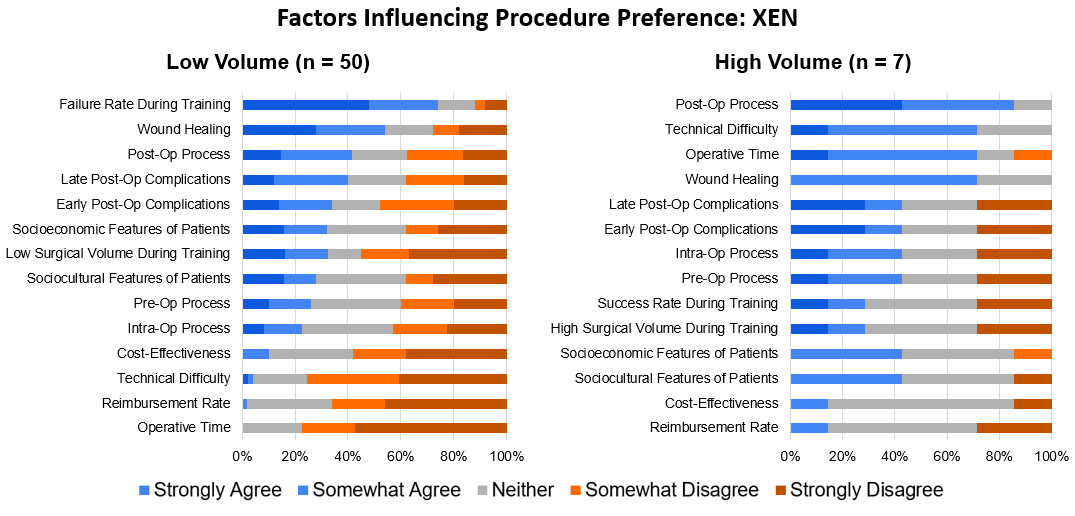


**(D) Ab-Interno Angle Procedure**


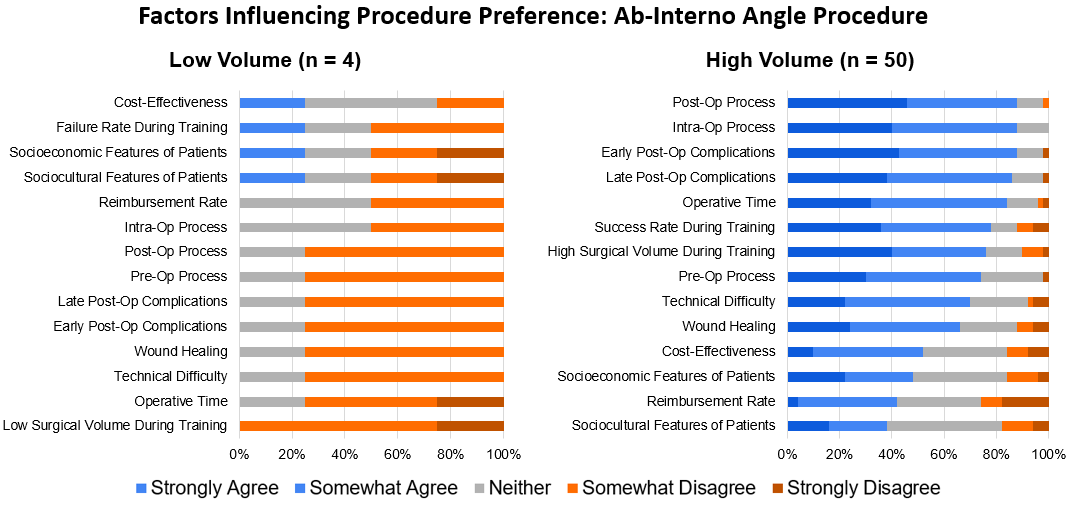

Supplement: Multimedia component 3 [file mmc3.docx]
